# Improved production of andrimid in *Erwinia persicina* BST187 strain by fermentation optimization

**DOI:** 10.1186/s12866-023-02946-2

**Published:** 2023-09-25

**Authors:** Tingfeng Cheng, Tongling Ge, Lunqiang Zhao, Yuyong Hou, Jianye Xia, Lei Zhao

**Affiliations:** 1 Key Laboratory of Engineering Biology for Low-carbon Manufacturing, Tianjin Institute of Industrial Biotechnology, Chinese Academy of Sciences, 32 West 7th Avenue, Tianjin Airport Economic Area, Tianjin, 300308 China; 2 University of Chinese Academy of Sciences, Beijing, China; 3 National Center of Technology Innovation for Synthetic Biology, Tianjin, China; 4 College of Biological Sciences, China Agricultural University, Beijing, China

**Keywords:** Andrimid, *Erwinia persicina*, Antibacterial activity, Fermentation, Design of experiment, Response surface methodology

## Abstract

**Background:**

Andrimid is reported to be a novel kind of polyketide-nonribosomal peptide hybrid product (PK-NRPs) that inhibits fatty acid biosynthesis in bacteria. Considering its great potential in biomedicine and biofarming, intensive studies have been conducted to increase the production of andrimid to overcome the excessive costs of chemosynthesis. In screening for species with broad-spectrum antibacterial activity, we detected andrimid in the fermentation products of *Erwinia persicina* BST187. To increase andrimid production, the BST187 fermentation medium formulation and fermentation conditions were optimized by using systematic design of experiments (One-Factor-At-A-Time, Plackett–Burman design, Response Surface Methodology).

**Results:**

The results indicate that the actual andrimid production reached 140.3 ± 1.28 mg/L under the optimized conditions (trisodium citrate dihydrate-30 g/L, beef extract-17.1 g/L, MgCl_2_·6H_2_O-100 mM, inoculation amount-1%, initial pH-7.0, fermentation time-36 h, temperature-19.7℃), which is 20-fold greater than the initial condition without optimization (7.00 ± 0.40 mg/L), consistent with the improved antibacterial effect of the fermentation supernatant.

**Conclusions:**

The present study provides valuable information for improving andrimid production via optimization of the fermentation process, which will be of great value in the future industrialization of andrimid production.

**Supplementary Information:**

The online version contains supplementary material available at 10.1186/s12866-023-02946-2.

## Background

As an essential enzyme that catalyzes the first committed step in fatty acid biosynthesis, which is responsible for the formation of malonyl-CoA from acetyl-CoA [1], acetyl-CoA carboxylase (ACC) is reported to be an effective target for antibacterial drug discovery due to the lack of primary sequence homology between the prokaryotic and eukaryotic forms [2, 3]. A pseudopeptide antibiotic named andrimid was detected with the ability to block the carboxyl-transfer reaction of bacterial ACC, resulting in the inhibition of fatty acid biosynthesis with submicromolar potency [2], which sheds light on the identification of new antibiotics against drug-resistant bacteria.

Andrimid was assigned to be a new class of natural antibiotics with polyketide–nonribosomal peptide hybrid products (PK–NRPs) and was initially identified in the culture broth of a symbiont of the brown planthopper *Nilaparvata lugens* approximately half a century ago [4]. It exhibits broad-spectrum antibacterial activity and is highly tolerant to high temperatures, strong acids and bases (unpublished results). *In silico* evaluation of andrimid as a novel antibiotic showed no systemic toxicity and the highest drug-likeness score (0.944) [5]. Thus, andrimid presents great potential as a selective antibiotic for widespread usage in both biomedicine and biofarming.

Andrimid has been widely detected in the culture broths of bacterial species, including *Pseudomonas fluorescens* [6], *Pantoea agglomerans* [7], *Serratia marcescens* [8], *Enterobacter cloacae* [9] and *Vibrio cholerae* [10]. Although the source of andrimid was studied, only a few fermentation strategies for andrimid production were mentioned in a previous study. Matilla et al. [11] reported that both temperature and carbon source affect andrimid biosynthesis in *S. plymuthica*, and enhanced antibiotic production was detected at lower temperatures in media containing citrate, gluconate or glycerol. Wietz et al. [12] found that chitin stimulates the production of andrimids in *V. coralliilyticus*. Sάnchez et al. [13] further confirmed that andrimid activity was detected only in cold-living *S. proteamaculans* cultured at low temperature (≤ 25℃). Unfortunately, the andrimid content of those strains is relatively low and time-consuming.

In screening for species with broad-spectrum antibacterial activity, we detected andrimid production in the fermentation products of *Erwinia persicina*, which belongs to the phylum Proteobacteria, class Gammaproteobacteria, order Enterobacteriales, family Enterobacteriaceae and genus *Erwinia* [14]. *E. persicina* exhibits great potential as a biological control agent to inhibit *Salmonella enterica* contamination in the sprout food industry [15], Fusarium head blight (FHB) severity in wheat [16] and a variety of soil-borne pathogens (*Alternaria solani*, *Sclerotinia sclerotiorum*, *Rhizoctonia solani* and *Fusarium proliferatum*) in potato dextrose agar plates [17]. Recently, *E. persicina* BST187 was isolated from the rhizosphere of tomato and exhibits broad-spectrum antibacterial activity due to its inhibitory effects on bacterial acetyl-CoA carboxylase. However, no systematic study of andrimid fermentation has been reported, which hinders its future industrialization.

To obtain higher yields of andrimid with the BST187 strain, both the fermentation media formulation and fermentation conditions could be optimized based on the andrimid-related gene clusters in BST187. Moreover, response surface methodology (RSM), which is known as Box-Wilson methodology [18] and recognized as an imperative technique for the optimization of specific responses influenced by variables, could be used in BST187 fermentation. A comparison between the Box-Behnken design (BBD) and other response surface designs has demonstrated that the BBD is more efficient than the central composite design, thus is has been widely used in completely randomized experiments [19]. Recently, RSM has been successfully used in various fields of microbial active products, such as optimizing the composition of medium and conditions for penicillin acylase and avermectin production by *Streptomyces* [20, 21].

The present study aims to increase andrimid production via optimization of the culture media composition and fermentation conditions of *E. persicina* BST187 using RSM. We investigated the effects of carbon source, nitrogen source, mineral salt, amino acid, initial pH, inoculation amount, fermentation temperature, fermentation time and dissolved oxygen content on andrimid production by one-factor-at-a-time (OFAT). Furthermore, Plackett‒Burman design (PBD) was applied to screen for significant parameters, which were optimized using BBD. We further confirmed the antibacterial effects of fermentation products of *E. persicina* BST187 under optimized conditions. Therefore, the present study provides important information for improving andrimid production, which will be of great value in the future industrialization of andrimid production.

## Result

### Establishment of the quantification methods of andrimid

**Fig. 1:**
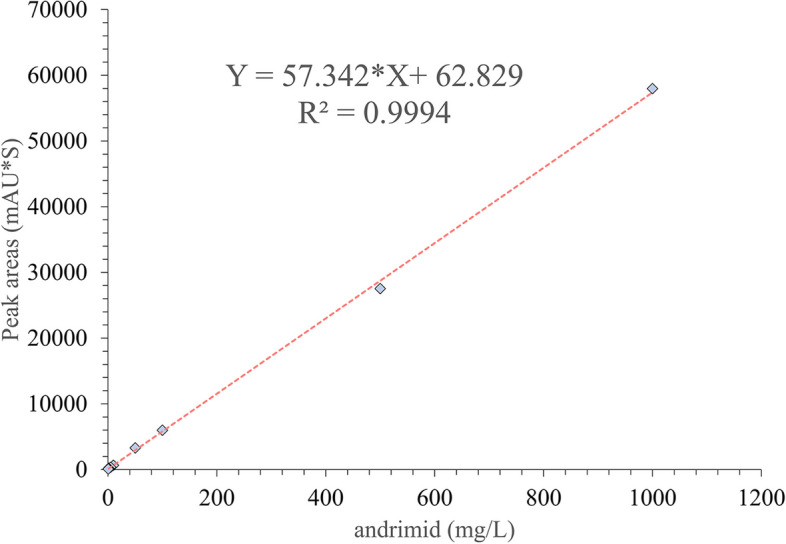
Calibration curve for andrimid quantification

Andrimid was reported to be purified and quantified in a previous study, and detailed information remains to be studied [22]. Here, we established quantification methods for andrimids by an external standards method. The purity of the andrimid was greater than 98%, according to the HPLC (high-performance liquid chromatography) profile (Fig. S1, Table S1). To quantify the concentration of andrimid in samples, we made a standard curve with the purified andrimid compound at eight concentration gradients. A high correlation was detected with the linearity of R^2^ > 0.9994 in the tested ranges (Fig. 1), which demonstrated the high accuracy of the developed method for the assay of andrimids generated by BST187 and used in the following experiments.

### Evaluation of BST187 growth and its andrimid production

**Fig. 2:**
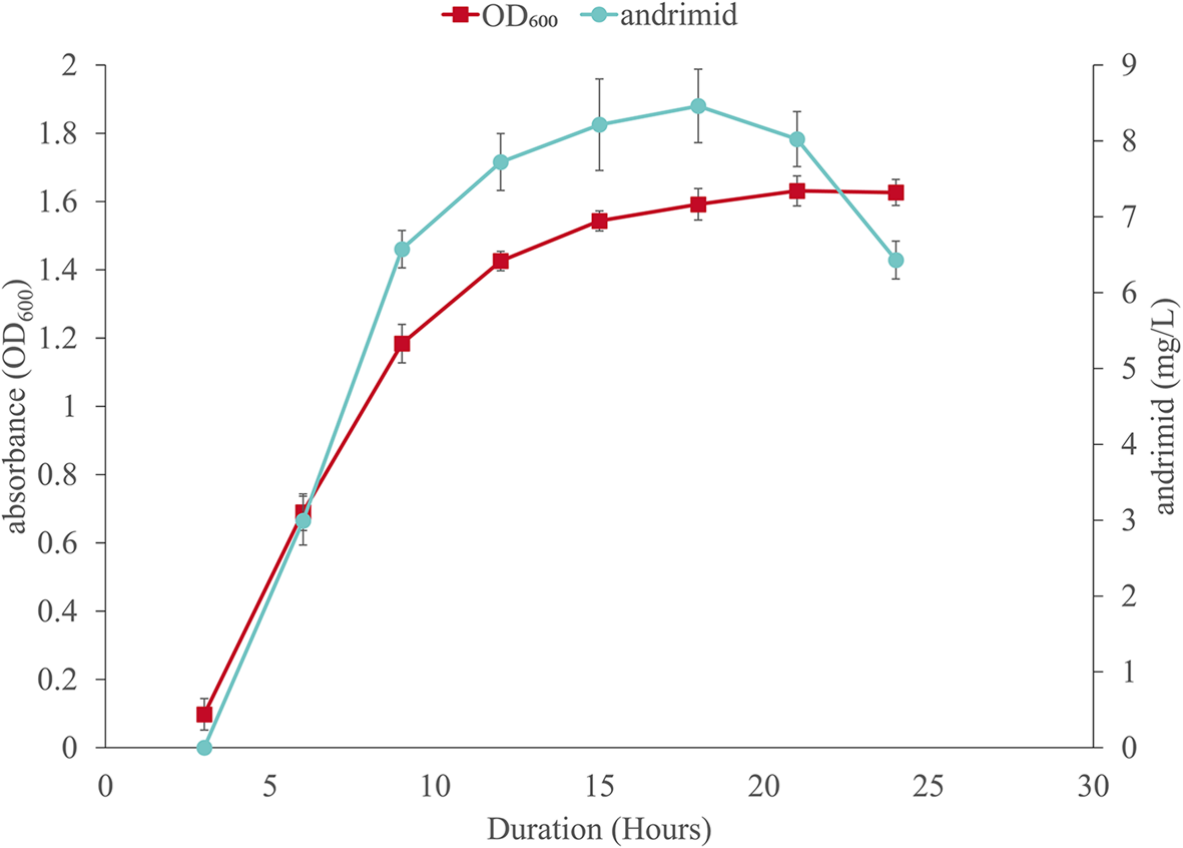
Growth curve and andrimid production of BST187 in shake flasks

To assess the growth of the BST187 strain, we initially grew the cells in LB (Luria-Bertani media). As shown in Fig. 2, BST187 cells were monitored with a fast growth rate and reached a lag phase at 3 h. After that, the bacteria entered the exponential growth phase, when the number of cells increased exponentially, with OD_600_ from 0.10 ± 0.05 at 3 h to 1.43 ± 0.03 at 12 h. After 12 h, the bacteria entered the stationary phase, and the cell proliferation rate was slower. Meanwhile, andrimid was generated and manifested as a positive relationship with the growth of BST187, resulting in a yield of 8.46 ± 0.49 mg/L at 18 h. However, the concentration of andrimid decreased quickly after the stationary phase.

### Effect of culture medium on andrimid production

To evaluate the effect of media composition on andrimid production, BST187 cells were grown in six culture media (Table S2). As indicated in Fig. S2, the highest biomass was detected when BST187 was cultured in KB (King’s B media), but little andrimid production was observed. In contrast, andrimid was assayed with a maximum value (13.51 ± 2.05 mg/L) when BST187 was cultivated in CB (Citrate-Beef media). Moreover, andrimid was not significantly different between 18 h (13.51 ± 2.05 mg/L) and 24 h (13.07 ± 2.65 mg/L).

### Optimization of BST187 fermentation conditions and andrimid production using the one-Factor-At-A-Time (OFAT) method

To optimize the medium formulation and fermentation conditions for BST187, OFAT was used to screen for the types and ranges of carbon source, nitrogen source, mineral salts, amino acids, initial pH, inoculation amount, fermentation time, fermentation temperature and dissolved oxygen content. The detailed information is as follows.

### Effect of carbon sources on andrimid production

**Fig. 3:**
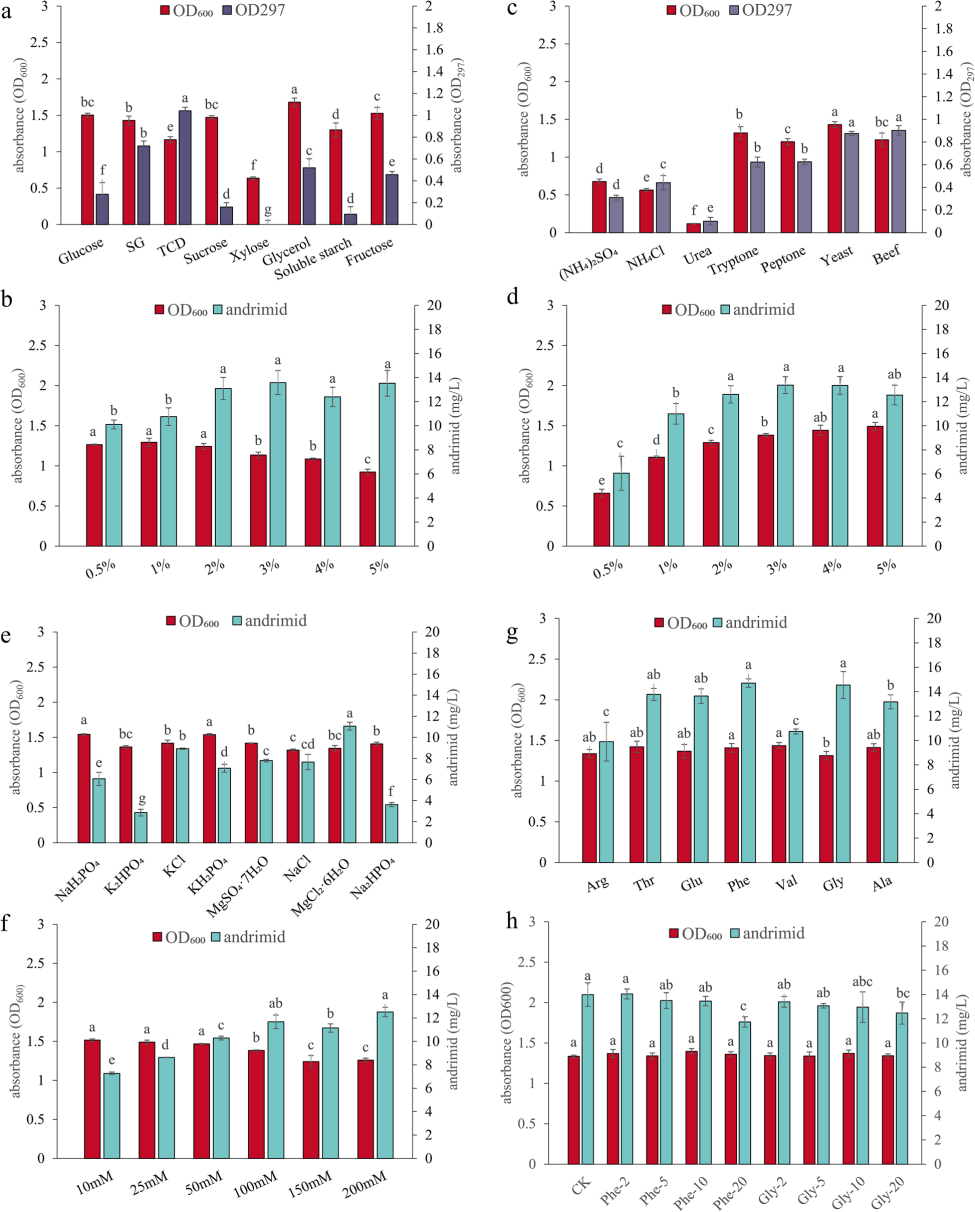
Effect of different types of carbon source (**a**), carbon source concentration (**b**), nitrogen source (**c**), nitrogen source concentration (**d**), mineral salt (**e**), mineral salt concentration (**f**), amino acid type (**g**), and amino acid concentration (**h**) on andrimid production of BST187. SG: sodium gluconate; TCD: trisodium citrate dihydrate; The different letters (a, b, c, d) represent the significant differences among the media (*p* < 0.05)

Based on the CB fermentation medium mentioned above, 2% of various carbon sources were added into the medium to evaluate their effects, including glucose, sodium gluconate, trisodium citrate dihydrate, sucrose, xylose, glycerol, soluble starch and fructose. The highest concentration of andrimid was detected when trisodium citrate dihydrate was used as the carbon source (Fig. 3a). The optimal amount of trisodium citrate dihydrate was further investigated with a series of concentrations, and a maximum value of 13.58 ± 1.00 mg/L andrimid was observed when 30 g/L trisodium citrate dihydrate was added (Fig. 3b). A high concentration of trisodium citrate dihydrate as a carbon source contributes to the production of andrimid from BST187, and the following optimizations are based on this formulation.

### Effect of nitrogen sources on andrimid production

Based on the optimized fermentation medium mentioned above, 1% of various carbon sources were added into the medium to evaluate their effects, including (NH_4_)_2_SO_4_, NH_4_Cl, urea, tryptone, peptone, yeast extract and beef extract. The highest concentration of andrimid was detected when beef extract was used as the nitrogen source (Fig. 3c). The optimal amount of beef extract was further investigated with a series of concentrations, and a maximum value of 13.37 ± 0.70 mg/L andrimid was observed when 30 g/L beef extract was added (Fig. 3d).

### Effect of mineral salts on andrimid production

Based on the optimized fermentation medium mentioned above, various mineral salts (100 mM) were added to the medium to evaluate their effects. The highest concentration of andrimid was detected when MgCl_2_·6H_2_O was used as the mineral salt (Fig. 3e). The optimal amount of MgCl_2_·6H_2_O was further investigated with a series of concentrations, and a maximum value of 12.51 ± 0.40 mg/L andrimid was observed when 200 mM MgCl_2_·6H_2_O was added (Fig. 3f). However, the concentration of andrimid was not significantly different between 100 mM (11.67 ± 0.57 mg/L) and 200 mM (12.51 ± 0.40 mg/L).

### Effect of amino acids on andrimid production

Based on the optimized fermentation medium mentioned above, the highest concentration of andrimid was detected when Phe (14.70 ± 0.35 mg/L) and Gly (13.63 ± 0.59 mg/L) were used as the amino acids (Fig. 3g). However, Phe and Gly did not significantly increase the concentration of andrimid compared with the control without amino acids added (Fig. 3h).

### Effect of inoculation amount on andrimid production

**Fig. 4:**
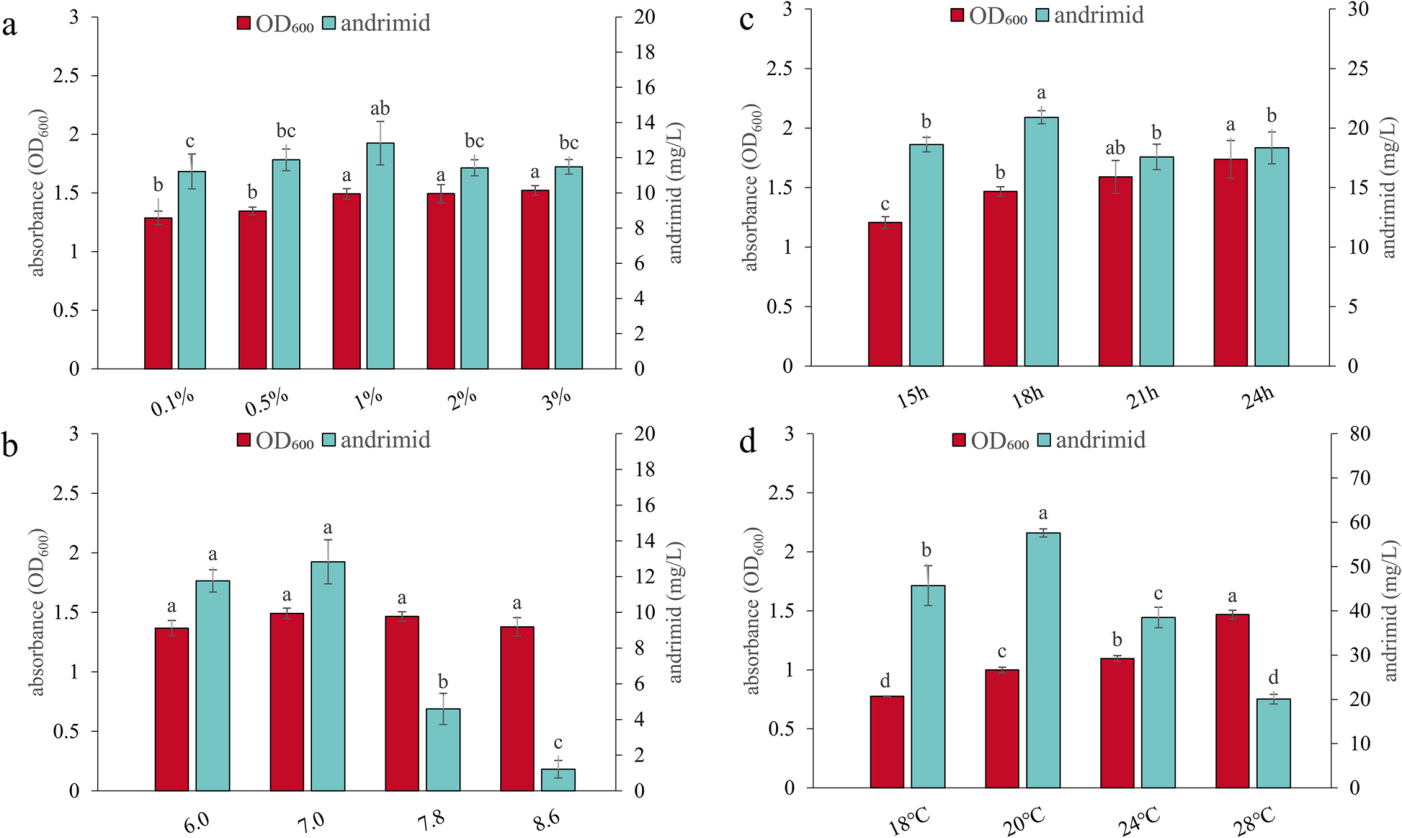
Effect of different initial pH (**a**), inoculation amount (**b**), fermentation time (**c**), and fermentation temperature (**d**) on andrimid production of BST187. The different letters (a, b, c, d) represent the significant differences among the media (*p* < 0.05)

Based on the CB fermentation medium mentioned above, the optimal inoculation amount was further investigated with a series of concentrations, and a maximum value of 12.82 ± 1.24 mg/L andrimid was observed when the inoculation amount was 1% (Fig. 4a). After that, the concentration of andrimid decreased with increasing inoculation amount.

### Effect of initial pH on andrimid production

Based on the CB fermentation medium mentioned above, the optimal initial pH was further investigated with a series of concentrations, and the concentration of andrimid was highest when the initial pH was 6–7 (Fig. 4b). After that, the concentration of andrimids significantly decreased with increasing pH, and there was no significant difference in biomass.

### Effect of fermentation time on andrimid production

Based on the optimized fermentation medium mentioned above, the fermentation time was further investigated from 15 to 24 h. The andrimid content gradually increased from 18.61 ± 0.61 mg/L in the initial 15 h of incubation to 20.90 ± 0.55 mg/L at 18 h (Fig. 4c). After that, the concentration of andrimids decreased with a significant increase in biomass.

### Effect of fermentation temperature on andrimid production

Based on the optimized fermentation medium mentioned above, the fermentation temperature was further investigated from 18 to 28 °C. The highest andrimid concentration (57.60 ± 0.92 mg/L) from BST187 was incubated at 20 °C compared with other temperatures, and biomass increased significantly with increasing temperature (Fig. 4d).

### Effect of dissolved oxygen content on andrimid production

Among the different dissolved oxygen (DO) contents screened, a high oxygen level was the most suitable for andrimid production by BST187. The andrimid concentration of BST187 reached a maximum value of 69.75 ± 1.64 mg/L when the conical flask was triple baffled. Furthermore, the biomass significantly increased from 1.09 ± 0.05 in the without baffled flask to 2.06 ± 0.03 in the triple baffled flask (Fig. S3b).

### Optimization of andrimid production using Response Surface Methodology (RSM)

#### Cultivation parameters screening

**Fig. 5:**
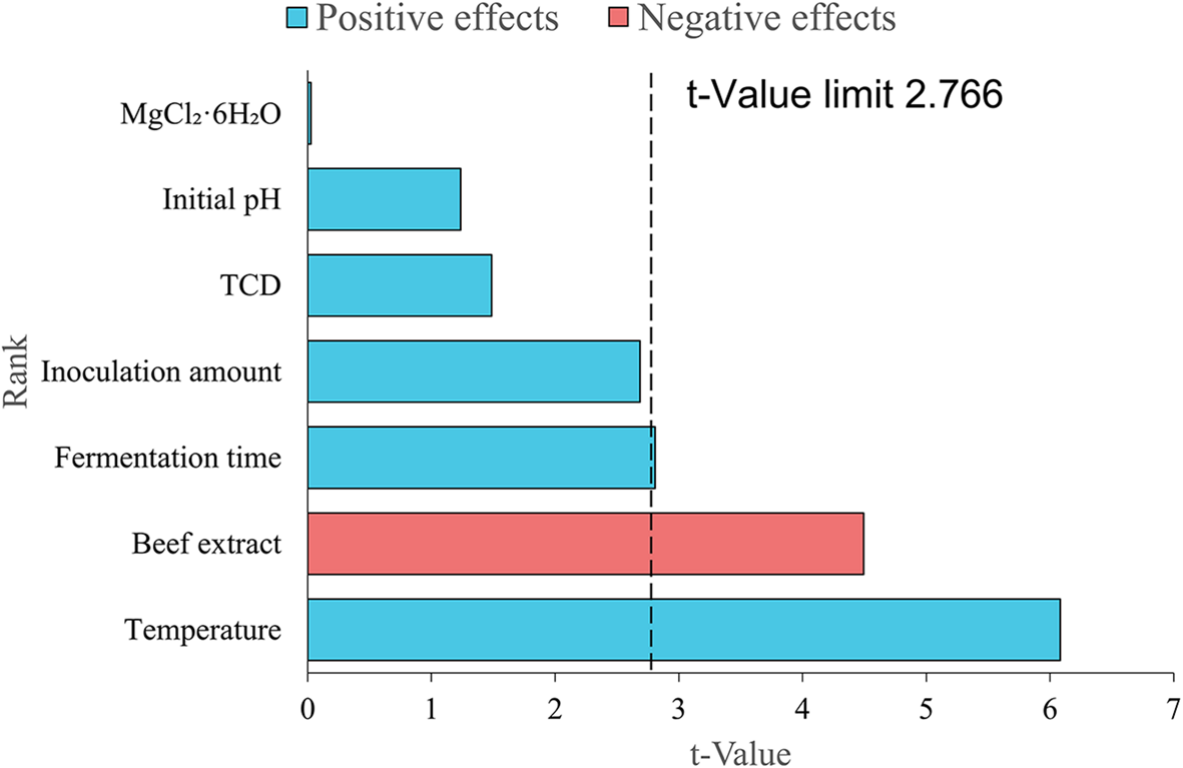
Pareto analysis of 7 influencing factors. TCD: trisodium citrate dihydrate

The optimization of cultivation parameters was conducted by using PBD experiments to determine the individual effects on andrimid production. The matrix design and actual response values for andrimid productivity are shown in Table S3, and the actual values of the process variables are listed in Table S4. According to PBD experiments, the influences of seven numeric factors on andrimid production were G (fermentation temperature) > B (beef extract) > F (fermentation time) > D (inoculation amount) > A (trisodium citrate dihydrate) > E (initial pH) > C (MgCl_2_·6H_2_O) (Fig. 5). Inasmuch as they were assayed with significant effects on andrimid production, thus were used as the main effect factors.

### Optimization of significant factors by Response Surface Methodology (RSM)

**Fig. 6:**
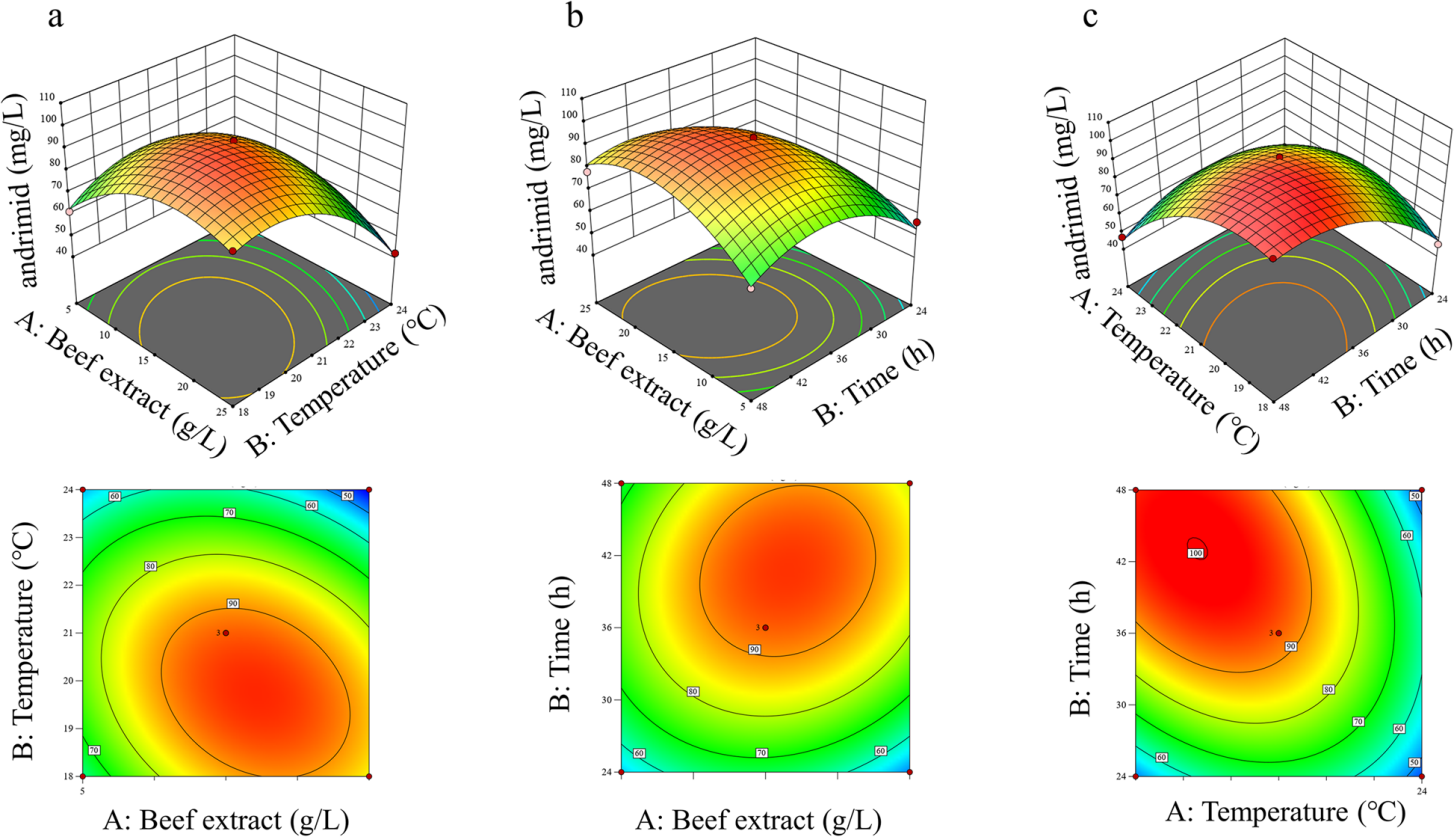
Response surface plot showing the interaction of different factors for andrimid production. **a** beef extract and fermentation temperature; **b** beef extract and fermentation time; **c** fermentation temperature and time

Based on the results above, BBD was further used to optimize G (fermentation temperature), B (beef extract) and F (fermentation time). The matrix design and actual response values for andrimid productivity are shown in Table S5. When the combination of A (trisodium citrate dihydrate)-30 g/L, B (beef extract)-15 g/L, C (MgCl_2_·6H_2_O)-100 mM, D (inoculation amount)-1%, E (initial pH)-7, F (fermentation time)-48 h and G (fermentation temperature)-18℃ was used for the medium, the highest andrimid production was obtained (up to 98.03 mg/L, Table S5). ANOVA (one-way analysis of variance) test indicates that the quadratic model fits the experimental data (*p* < 0.01) and has high model accuracy (*F* value of 67.3). The model terms B, C, AB, AC, BC, A², B² and C² had significant effects on andrimid content (Table 1, *p* < 0.05). According to the ANOVA, temperature and beef extract were detected with the highest effect (*p* < 0.0001) and lowest effect (*p* = 0.0624) on the response, respectively. This result is consistent with OFAT analysis in which temperatureis observed with strong influenced on andrimid. The interactive effect of variables AB (beef extract and fermentation temperature) and BC (fermentation temperature and time) had the most impact on andrimid content (Fig. 6a and b) compared to AC (beef extract and fermentation time) (Fig. 6c). The second-order polynomial equation of andrimid (Y) is given below:

**Table 1:** ANOVA for Quadratic Model (Response: andrimid content)

Source	Sum of Squares	df	Mean Square	F-value	*p*-value
Model	6035.7	9	670.63	67.3	0.0001
A-Beef extract	56.91	1	56.91	5.71	0.0624
B-Temperature	1465.88	1	1465.88	147.09	< 0.0001
C-Fermentation time	1052.68	1	1052.68	105.63	0.0001
AB	268.93	1	268.93	26.99	0.0035
AC	87.24	1	87.24	8.75	0.0316
BC	583.26	1	583.26	58.53	0.0006
A²	791.16	1	791.16	79.39	0.0003
B²	1222.31	1	1222.31	122.65	0.0001
C²	888.81	1	888.81	89.19	0.0002
Residual	49.83	5	9.97		
Lack of Fit	43.38	3	14.46	4.48	0.1878
Pure Error	6.45	2	3.23		
Cor Total	6085.53	14			
Std.Dev.	3.16				
Mean	67.06				
C.V.%	4.71				
PRESS	705.54				
R^2^	0.9918				
Adjusted R^2^	0.9771				
Predicted R^2^	0.8836				
Adeq Precision	21.4447

$$\mathrm{Andrimid}(\mathrm Y)=92.85+2.67\ast\mathrm A-13.54\ast\mathrm B+11.47\ast\mathrm C-8.20\ast\mathrm{AB}+4.67\ast\mathrm{AC}-12.08\ast\mathrm{BC}-14.64\ast\mathrm A^2-18.19\ast\mathrm B^2-15.52\ast\mathrm C^2$$

The expected andrimid contents were derived using regression analysis and compared to experimental data, indicating that the projected response values were consistent with the actual response values (Fig. S4). According to the regression analysis results, although the content of andrimid still showed an increase with fermentation time, the increase was less than that in the previous period, and 36 h was chosen as the final fermentation time based on cost considerations (Fig. 6c). In order to determine the optimal values to maximize andrimid content, a numerical hill climbing technique was used to find the extreme point of the model. Table 2 shows the suggested solution for the constraint of beef extract (17.1 g/L), temperature (19.7℃) and fermentation time (36 h) to maximize andrimid content (95.96 mg/L).

**Table 2:** Constraints, criteria and solution for optimization of andrimid production

Name	Goal	Low limit	Upper limit	Importance	Solution
A (g/L)	In range	5	25	3	17.09
B (℃)	In range	18	24	3	19.74
C (hours)	Target	36	36	3	36
andrimid mg/L	Maximize	42.08	98.025	3	95.963

A Beef extract; B Temperature; C Fermentation time

### Andrimid production in the optimized conditions

With the optimized conditions, andrimid was detected at 92.43 ± 3.69 mg/L in the optimized CB medium, which was close to the predicted value of 95.96 mg/L. Furthermore, combined with high oxygen, the andrimid production reached 140.3 ± 1.28 mg/L, which is improved by 20 times of the initial fermentation (Table 3).

**Table 3:** Yield of andrimid in tested strains and culture conditions reported here and previous studies

Medium	Culture condition	Strain	Standard	Yield (mg/L)	Reference
TSB	5 day, 24℃	*Enterobacter* sp.	Flash Chromatography	1.22	[1]
TSB (solid)	3 day, room temperature	*Pseudomonas fluorescens*	Flash Chromatography	43.48	[6]
2xYT	1 day, 28℃	*Pantoea agglomerans*	-^a^	378.16	[22]
LB	7 day, 8℃	*Serratia proteamaculans*	Semi-preparative RP-HPLC	4.62	[13]
LB	18 h, 28℃	*Erwinia persicina*	Preparative HPLC	7.00 ± 0.40	This study
CB	36 h, 19.7℃	*Erwinia persicina*	Preparative HPLC	92.43 ± 3.69	This study
CB	36 h, 19.7℃, baffled flask	*Erwinia persicina*	Preparative HPLC	140.31 ± 1.28	This study

^a^Andrimid were determined by a LC-MS assay with standards at known concentrations (details of the standards are not mentioned)

### The antibacterial assay of andrimid

**Fig. 7:**
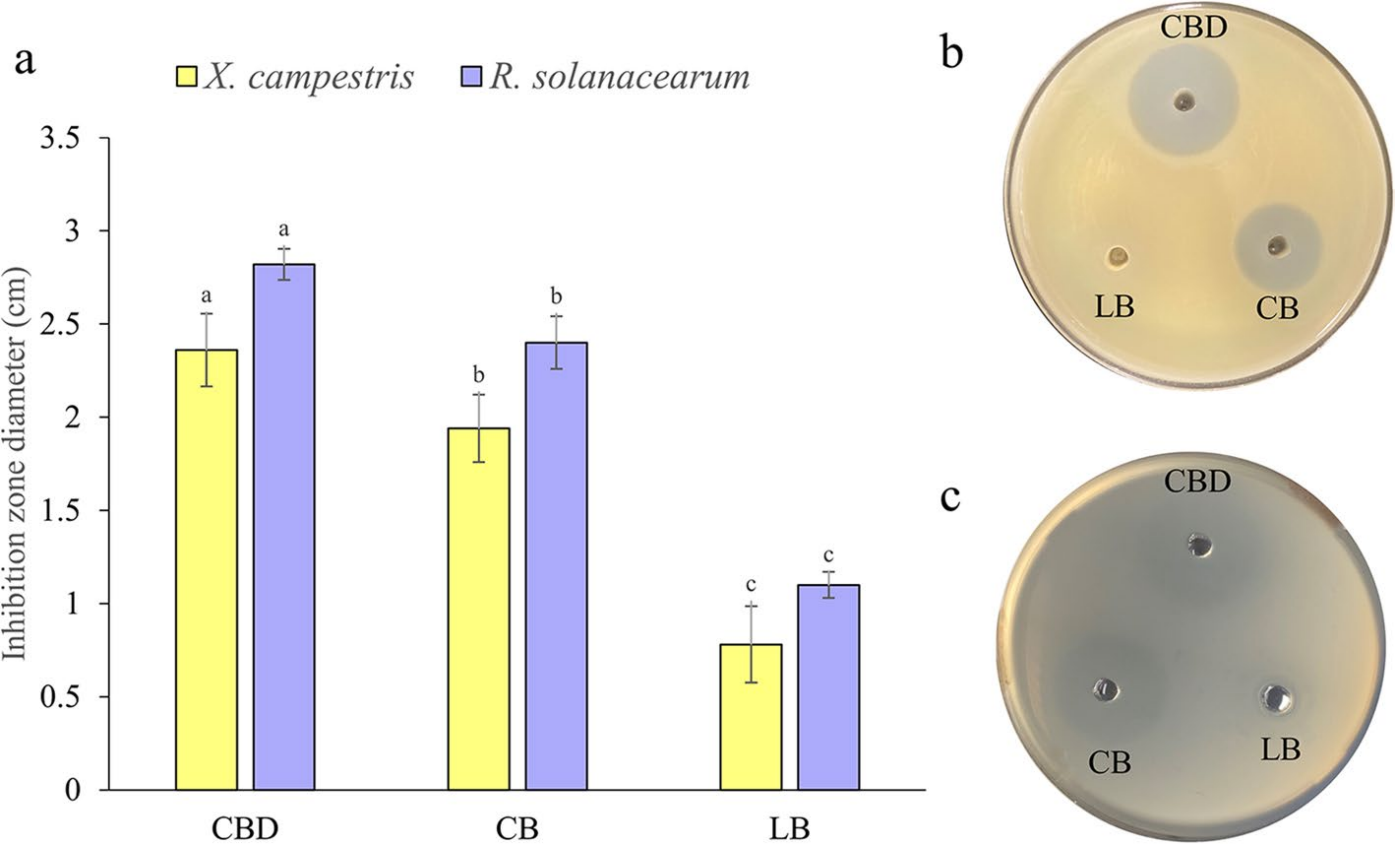
Inhibitory effects of andrimid from different fermentation media and fermentation conditions. **a** Inhibitory effects of andrimid; LB: Luria–Bertani; CB: optimized Citrate-Beef media; CBD: optimized Citrate-Beef media combined with high oxygen. The different letters (a, b, c, d) represent the significant differences among the media (*p* < 0.05); **b** *Xanthomonas campestris* pv. *Vesicatoria*; **c** *Ralstonia solanacearum*

The antibiotic activity results showed that the inhibitory effect of the BST187 supernatant was significantly improved (Fig. 7a). The inhibition zone diameter of the optimized CB medium (baffled flask) against *X. campestris* pv. *vesicatoria* reached 2.36 ± 0.79 cm (Fig. 7b). Similarly, the antibacterial effect of the fermentation supernatant on *R. solanacearum* was observed when BST187 was cultured in optimized CB medium (Fig. 7c). The inhibition zone diameters ranged from 1.10 ± 0.07 cm (LB) to 2.82 ± 0.07 cm (optimized CB, baffled flask).

## Discussion

The development and spread of multidrug-resistant bacteria are great challenge to humankind in both global health and agricultural production, which urgently requires the discovery of novel bioactive and natural products [23–25]. We initially sought to identify antagonistic bacteria by using a reporter strain, with the subsequent aim of testing whether these strains could be used for biological control in agriculture. This strategy resulted in the discovery of the ACC inhibitor andrimid produced by *E. persicina* BST187 (submitted manuscript). Andrimid and moiramide B are members of pyrrolidine dione antibiotics family. Transcriptome analyses with *Bacillus. subtilis* revealed that the initial biosynthesis of fatty acid was transcriptionally down-regulated when adding moiramide B [26]. Resistance mutations induced by pyrrolidine diones are exclusively located in the carboxyltransferase subunits of the bacterial ACC [27]. Therefore, andrimid acts as an ACC inhibitor targets the carboxyltransferase reaction of this enzyme with a competitive inhibition pattern versus malonyl-CoA [28]. In this study, we systematically optimized both the fermentation formula and conditions for BST187, leading to a 20-fold improvement in andrimid production. Our results highlight the potential industrialization and application of andrimid in the future via fermentation modification.

### Establishment of andrimid quantification

Although andrimid was identified more than 30 years ago, a vast majority of studies have focused on its chemosynthesis and the functional dissection of related gene clusters. The high cost and environmental pollution of chemosynthesis has hindered the potential application of andrimid [29, 30], and the biosynthesis approach is emerging as a promising method with the development of metabolic engineering [31].

Andrimid is reported to be detected in the culture broths of bacteria, including *Pseudomonas fluorescens* [6], *Pantoea agglomerans* [7], *Serratia marcescens* [8], *Enterobacter cloacae* [9] and *Vibrio cholerae* [10], but the corresponding fermentation is poorly studied due to the low production of andrimid, lack of andrimid standard and quantification method [32, 33]. In this study, we found that the absorbance at OD_297_ could be used for preliminary screening of the concentration of andrimid. Subsequently, we used purified standards enabling the absolute quantification of andrimids with the newly isolated BST187 strain, which confirmed the assay of andrimids used in fermentation optimization.

### Optimization of fermentation medium formulation revealing their effects on andrimid production

With the identified BST187 strain, we further optimized the fermentation medium to increase andrimid production. The classical OFAT has been widely used in medium optimization for ease and convenience [34]. Here, we successfully screened a suitable medium composition for the BST187 strain and obtained high levels of andrimid production.

#### Carbon source

The type of carbon source is the main factor for cell growth as well as the production of primary and secondary metabolites [34]. We revealed that 3% trisodium citrate dihydrate is favorable to increase andrimid through OFAT analysis, which is consistent with a previous study in which the rhizosphere bacteria *S. plymuthica* A153 exhibited maximal antibacterial activity in the presence of citrate [11]. In fact, the roots exude considerable amounts (11–40%) of photosynthetically fixed carbon, including sugars, organic acids and amino acids [35, 36]. BST187 was also isolated from the rhizosphere with different environmental carbon sources, which is beneficial in maintaining the capacity of BST187 to overcome bacterial competitors in its natural niche [11, 37].

#### Nitrogen source

Nitrogen is involved in the biosynthesis of secondary metabolites, and cell growth is limited by nitrogen sources [38]. The beef and yeast extracts significantly increased the andrimid concentration in the fermentation broth of strain BST187 through OFAT analysis. Organic beef extract contains a wide variety of proteins, inorganic salts, vitamins, and growth-promoting factors that are more nutritious and promote greater protease production than other nitrogen sources [39].

#### Mineral salts

Mineral salts are another basic component that are critical for the stabilization of membranes and ribosomes and the neutralization of nucleic acids [40]. The OFAT analysis showed that MgCl_2_·6H_2_O as the source of mineral salt increased the andrimid concentration in the fermentation broth of BST187. Organisms must maintain physiological levels of Mg^2+^ because this divalent cation is critical as a cofactor in a variety of enzymatic reactions, including ACC, which confers resistance in the andrimid producer [2, 40].

#### Amino acids

The amino acid composition in fermentation media is important because deficiencies in these nutrients reduce desired yields, and unsuitable amino acids may inhibit the synthesis of secondary metabolites [41]. For amino acids, the andrimid concentration of BST187 was higher than the others when phenylalanine and glycine were used. However, the increase in amino acid concentration reduces the content of andrimid. Actually, the andrimid chemical precursor contains phenylalanine, valine and glycine, and they can be synthesized by itself to meet the requirements of andrimid synthesis [7].

In summary, an appropriate concentration of carbon source trisodium citrate dihydrate, organic nitrogen beef extract and mineral salts MgCl_2_·6H_2_O are favorable to increased andrimid in BST187. This is mainly due to their unique roles in andrimid biosynthesis; for example, *V. coralliilyticus* S2052 synthesized higher levels of andrimid with both chitin and macroalgal extracts (abundant in the marine environment) as the sole nutrient source [12], while BST187 and *S. plymuthica* A153 from the rhizosphere produced more andrimid with citrate as the sole carbon source. The substrate preference of BST187 suggests an important ecophysiological role of andrimid that provides plant pathogens with an advantage against bacteria, ultimately enhancing their ability to infect plants [42, 43].

### Optimization of fermentation conditions enhances andrimid production

Fermentation conditions are one of the key processes determining the overall economics of bioprocesses in industrial scale production [44]. With the optimized fermentation medium formulation, we further optimized the fermentation conditions aiming to increase andrimid production. In this study, the fermentation conditions of initial pH, inoculation amount, temperature, time and dissolved oxygen content were optimized using OFAT experiments, and that temperature, time and dissolved oxygen content significantly affected the andrimid concentration in the fermentation broth of BST187.

#### Fermentation temperature and time

Bacteria can sense and respond to temperature by regulating temperature-dependent gene expression [45], and the effect of temperature on andrimid production has been reported in previous studies [12, 13]. Here, we found that there was a gradual increase in the production of andrimids as the temperature decreased (20 °C vs. 28 °C; Fig. 4d), which is consistent with a previous study in which andrimid production was decreased at higher temperatures in several strains (*Vibrio cholerae*, *S. plymuthica*) [11, 46].

Furthermore, the andrimid content gradually increased in the exponential phase (Fig. 2). Transcriptome analysis also showed that andrimid biosynthetic genes were significantly upregulated only in the exponential phase [33]. However, along with prolongation of the culture time, the concentration of andrimid decreased steadily, possibly because andrimid was used as a carbon source and nutrient source by bacteria.

#### Dissolved oxygen

DO level is an important factor in aerobic fermentation that could significantly influence bacterial metabolism and product yield, and optimization of DO concentration is always necessary for industrial bioprocesses [47]. BST187 produced higher levels of andrimid in the 250 mL shake flask than in the 50 mL shake flask through OFAT analysis. Similarly, andrimid production increases in the baffled flask, and the presence of the baffled increases the gas-liquid oxygen transfer capacity inside the flask compared with the unbaffled flask [48]. This main reason may be that high oxygen could increase biomass [49]. Furthermore, the activity of the enzyme responsible for andrimid synthesis could be increased under high dissolved oxygen content.

In summary, temperature, time and dissolved oxygen content significantly increased the andrimid concentration from BST187. This mainly results from the expression changes of the andrimid biosynthetic gene clusters, which is supported by a previous study in which temperature regulated the andrimid gene cluster and no expression was observed above 30 °C [11]. The environmental suitability identified here may suggest the increased competitiveness of BST187 in conditions mimicking natural conditions [46].

### Combination of optimized medium formulation and fermentation conditions resulting in the high yield of andrimid production by BST187

Although both the medium formulation and fermentation conditions had been optimized by OFAT, their interaction remained to be determined because only a single factor was considered in the analysis. PBD, as a two-level design, is very useful for economically detecting their main effects [46]. Fermentation temperature, beef extract and fermentation time were revealed to be significant influences of factors on andrimid production with PBD experiments, which was consistent with previous studies that temperature and time significantly increased andrimid concentration [11].

Then, BBD in RSM was employed to evaluate the influence of process variables on andrimid production and predict the most relevant response for the highest yield. Maximum andrimid production was observed in the middle levels of beef extract (Fig. 6a), while excess levels resulted in a gradual decrease in andrimid production. There was a delay in the fermentation time to reach the highest andrimid production as the temperature decreased, however, a longer fermentation time increased the cost. Here, we determined that the highest production occurred at 19.7 °C when 36 h was the target time. Based on the model analysis, the maximum production of andrimid (95.96 mg/L) could be reached under the following conditions: A (trisodium citrate dihydrate)-30 g/L, B (beef extract)-17.1 g/L, C (MgCl_2_·6H_2_O)-100 mM, D (inoculation amount)-1%, E (initial pH)-7, F (fermentation time)-36 h and G (fermentation temperature)-19.7℃. The actual values (92.43 ± 3.69 mg/L) were closer to the above predicted values, indicating that the predicted model agrees well with the actual situation. With the optimized medium combined with high oxygen (culture in a baffled flask), andrimid production reached 140.3 ± 1.28 mg/L, as confirmed by the increased antibacterial activity. Therefore, andrimid production was increased by 20-fold using RSM-optimized medium and conditions in comparison to the original culture method. Furthermore, andrimid production was achieved at a relatively high yield in a shorter time compared to a previous study (Table 3), which will be of great value in the future industrialization of andrimid production.

## Conclusion

This study presents the optimization of fermentation medium formulation and fermentation conditions of andrimids from *Erwinia persicina* using a systematic design of experiments (OFAT, PBD, RSM) for the first time to our knowledge. With the developed parameters, the maximum andrimid yield is expected to reach 95.96 mg/L, which is in agreement with the actual value of 92.43 ± 3.69 mg/L. The andrimid production was further increased to 140.3 ± 1.28 mg/L with higher dissolved oxygen in the baffled flask, resulting in a value 20-fold greater than the initial value without optimization (7.0 ± 0.40 mg/L). Therefore, the present study provides valuable information for improving andrimid production, which will be used for its industrialization. Furthermore, the results suggest an important ecophysiological role of andrimids that influences interspecific interactions between bacteria at the microscale. This study provides a strong basis for future practical extension to BST187 ecosystems and large-scale industrial production.

## Materials and methods

### Strains and culture conditions

The strain *E. persicina* BST187 was isolated from the rhizosphere soil of tomato and maintained in our laboratory. The strain was deposited in the China Center for Type Culture Collection (CCTCC) with accession number CCTCC M 2,022,872. Two plant pathogens, *Ralstonia solanacearum* and *Xanthomonas campestris pv. vesicatoria* were kindly provided by Dr. Laixin Luo from China Agricultural University and stored at -80 °C in our laboratory.

All strains were cultivated in Luria-Bertani media (10 g/L NaCl, 10 g/L tryptone and 5 g/L yeast extract) overnight at 28 °C with an agitation speed of 200 rpm.

### Determination of biomass and andrimid production

The biomass of BST187 was characterized by optical density (OD) at 600 nm. Briefly, 1 mL of sample was collected and then centrifuged at 13,000 rpm for 5 min. Cells were then resuspended, and 200 µl of cell resuspension was used to measure the OD_600_ by a 96-well microplate reader (SpectraMax M2e, Molecular Devices, CA, USA). The reserved supernatant was used for the determination of andrimid production.

The andrimid production was characterized by absorbance at 297 nm and reversed-phase high-performance liquid chromatography (RP-HPLC). For the initial assay of andrimid production, 200 µl of the above reserved supernatant was used to preliminarily determine the concentration of andrimid by measuring the absorbance at 297 nm. For the accurate quantification of andrimid, the fermentation supernatant was filtered through a 0.22 μm nylon filter and injected into the HPLC system. Chromatography was performed on an Agela & Phenomenex Innoval C18 column (4.6 × 250 mm; 5 μm; 100 A) with the Agilent 1260 Infinity II HPLC-DAD system (Agilent Technologies, Palo Alto, CA, USA). The injection volume was 10 µL, and the column oven was maintained at 30 °C. Andrimids were well-separated sequentially under the following isocratic elution conditions: mobile phase A, water + 0.1% formic acid; mobile phase B, methanol + 0.1% formic acid; 30% mobile phase A run time was 15 min at a flow rate of 0.7 mL/min and detection wavelength was set at 297 nm.

### Estimation of andrimid contents using external standards

The standard of andrimid (powder, 98% purity) was previously prepared in our laboratory (submitted manuscript) and was used to build the calibration curve between the peak area and concentration of andrimid (Table S1). Briefly, the pure products were weighed precisely and dissolved in water:methanol (1:1). Then, the original solution was diluted into a series of standard solutions with gradient concentrations (1000, 500, 100, 50, 10, 5, 1 and 0.5 mg/mL). Peak purity and areas of andrimid were determined by HPLC.

### Optimization of andrimid production using the one-Factor-At-A-Time (OFAT) method

To optimize the fermentation conditions of the BST187 strain, various parameters (carbon source, nitrogen source, mineral salt, amino acid, initial pH, inoculation amount, fermentation time, fermentation temperature and dissolved oxygen content) were studied using the OFAT method as described in the Supplemental Methods. All experiments were repeated at least three times.

### Optimization of andrimid production using Response Surface Methodology (RSM)

#### Selection of significant variables for andrimid production by Plackett–Burman design

The Plackett–Burman statistical design is an efficient method for screening and identifying the significant parameters among a large number of variables [50]. The main factors were screened based on seven factors (carbon source, nitrogen source, mineral salt, initial pH, inoculation amount, fermentation temperature and fermentation time), and the contents of andrimid (Y) were taken as the response value. To screen out the most important factors, two levels were taken for each factor, named “1” and “-1”. The Plackett‒Burman experimental design and response value are shown in Tables S3 and S4.

#### Box–Behnken design and response surface analysis

In the response surface analysis, the effects of culture medium and fermentation conditions on andrimid production were explored using a BBD with three factors (beef extract, fermentation temperature, fermentation time) and three levels (− 1, 0, 1) (Table S5). The experimental design and statistical analysis were carried out using Design-Expert software (Stat-Ease Inc., Minneapolis, MN, USA), with a total of 15 sets of experiments, including three center points. The andrimid concentration was calculated and used as the actual response. In addition, predicted response values were calculated using the regression equation.

#### Concentration of andrimid in optimized conditions

To measure the concentration of andrimid under optimized conditions, BST187 was cultured in optimized CB medium (30 g/L trisodium citrate dihydrate, 17.1 g/L beef extract, 100 mM MgCl_2_·6H_2_O, initial pH 7) and inoculated with 1% inoculum. Flasks were incubated on a rotary shaker (200 rpm) at 19.7 ± 1 °C for 36 h.

#### The antibacterial assay of andrimid

The antibacterial effect was tested using revision agar lawn assays. Briefly, 100 mL LB indicator plates (1.5% agar) containing 1 mL of an overnight culture of the bacterial strain were used for testing. BST187 bacterial cells were pelleted by centrifugation (13,000 rpm, 5 min), and the fermentation supernatant was filtered through a 0.22 μm nylon filter. Finally, 80 µl of the filter-sterilized supernatant was added to the punched-hole LB indicator plates and incubated at 28 °C for 48 h [11]. In addition, the andrimids were determined using the fermentation supernatant by HPLC. All experiments were repeated at least five times.

### Statistical analysis

Statistical analysis was performed using Statistical Product and Service Solutions 22.0 (IBM, Chicago, IL, USA). The results are indicated as the mean ± SE. Statistical significance was determined by ANOVA and the post hoc least square difference (LSD) test. Differences were considered significant at *p* < 0.05.

### Supplementary Information

**Additional file 1: Table S1.** The peak area of andrimid standard solution. **Table S2.** Composition of the candidate media evaluated within this study. **Table S3.** Plackett-Burman experimental design and response value. **Table S4.** Results of Plackett-Burman experiment design. **Table S5.** Design and results of Box-Behnken experiment. **Figure S1.** Analysis of HPLC chromatograms: standard of andrimid. **Figure S2.** The biomass and andrimid production of BST187 in tested media. **Figure S3.** Effect of dissolved oxygen content on andrimid production. **Figure S4.** The correlation between the predicted and actual values for andrimid.

## Data Availability

The original contributions presented in the study are included in the article. Further inquiries can be directed to the corresponding author.

## Abbreviations

ACC: Acetyl-CoA carboxylase
PK–NRPs: Polyketide–nonribosomal peptide hybrid products
FHB: Fusarium head blight
OFAT: One-factor-at-a-time
PBD: Plackett‒Burman design
BBD: Box‒Behnken design
RSM: Response surface methodology
HPLC: High-performance liquid chromatography
RP-HPLC: Reversed-phase high-performance liquid chromatography
LB: Luria-Bertani media
DO: Dissolved oxygen
CCTCC: China Center for Type Culture Collection
OD: Optical density
ANOVA: One-way analysis of variance
LSD: Least square difference
KB: King’s B media
CB: Citrate-Beef media
SG: Sodium gluconate
TCD: Trisodium citrate dihydrate
