# Supplementary material for: Improved production of andrimid in Erwinia persicina BST187 strain by fermentation optimization
Source: BMC Microbiol. 2023 Sep 25;23:268. doi: 10.1186/s12866-023-02946-2 (PMC10519088; doi:10.1186/s12866-023-02946-2)
Supplement: Supplementary file 1 — Additional file 1: Table S1. The peak area of andrimid standard solution. Table S2. Composition of the candidate media evaluated within this study. Table S3. Plackett-Burman experimental design and response value. Table S4. Results of Plackett-Burman experiment design. Table S5. Design and results of Box-Behnken experiment. Figure S1. Analysis of HPLC chromatograms: standard of andrimid. Figure S2. The biomass and andrimid production of BST187 in tested media. Figure S3. Effect of dissolved oxygen content on andrimid production. Figure S4. The correlation between the predicted and actual values for andrimid. [file 12866_2023_2946_MOESM1_ESM.docx]

***BMC Microbiology***

**Improved production of andrimid in *Erwinia persicina* BST187 strain by fermentation optimization**

Tingfeng Cheng^1,2^, Tongling Ge^1,4^, Lunqiang Zhao^1,3^, Yuyong Hou^1,4^, Jianye Xia^1,4*^, Lei Zhao ^1,3,4*^

^1^ Key Laboratory of Engineering Biology for Low-carbon Manufacturing, Tianjin Institute of Industrial Biotechnology, Chinese Academy of Sciences, Tianjin, China

^2^ University of Chinese Academy of Sciences, Beijing, China.

^3^ College of Biological Sciences, China Agricultural University, Beijing, China

^4^ National Center of Technology Innovation for Synthetic Biology, Tianjin, China

* Correspondence:

Lei Zhao,

Key Laboratory of Engineering Biology for Low-carbon Manufacturing, Tianjin Institute of Industrial Biotechnology, Chinese Academy of Sciences

32 West 7th Avenue, Tianjin Airport Economic Area, Tianjin 300308, China

Email: [zhaol@tib.cas.cn](mailto:zhaol@tib.cas.cn)

Jianye Xia,

Key Laboratory of Engineering Biology for Low-carbon Manufacturing, Tianjin Institute of Industrial Biotechnology, Chinese Academy of Sciences

32 West 7th Avenue, Tianjin Airport Economic Area, Tianjin 300308, China

Email: [xiajy@tib.cas.cn](mailto:xiajy@tib.cas.cn)

**Supplemental Methods**

**Determination of the BST187 strain growth curve**

A single colony of the BST187 strain was picked from an LB agar plate and inoculated into a 50 mL centrifuge tube (sterile) containing 30 mL LB liquid medium. Cells were incubated at 28°C for 8-12 h under shaking at 200 rpm, and the resulting culture was used as seed culture. Then, a 1 mL seed culture was transferred into a 250 mL flask containing 100 mL LB medium and incubated at 28°C for 24 h under shaking at 200 rpm. One microliter of bacterial suspension was collected by centrifugation for 5 min at 13000 rpm every 3 h from 0 to 24 h. The biomass and andrimid were evaluated by OD_600_ and HPLC, respectively. All experiments were repeated at least three times.

**Evaluation of the effect of basal media**

A 1% (v/v) aliquot of the BST187 seed culture was transferred into a 50 mL flask containing 30 mL fermentation culture using six different media as described in Table S2 and incubated at 28°C for 18 h and 24 h under shaking at 200 rpm. The medium was prepared using double distilled water (ddH_2_O) and sterilized at 121°C for 15 min. The andrimids were determined using the fermentation supernatant by HPLC, and the highest andrimid yield was used as the optimal basal medium. Subsequent optimization was carried out based on the optimal basal medium for andrimid production.

**Optimization of andrimid production using the** **One‑Factor‑At‑A‑Time (OFAT) Method**

To optimize the fermentation conditions of the BST187 strain, various parameters (carbon source, nitrogen source, mineral salt, amino acid, initial pH, inoculation amount, fermentation time, fermentation temperature and dissolved oxygen content) were studied using the One‑Factor‑At‑A‑Time (OFAT) method as described in the following sections. All experiments were repeated at least three times.

**Screening of fermentation medium formulation to maximize andrimid production**

Carbon source: 30 mL of media was prepared in 50 mL conical flasks supplemented with 2% of various carbon sources (glucose, sodium gluconate, trisodium citrate dihydrate, sucrose, xylose, glycerol, soluble starch and fructose). Cells were then incubated at 28°C and shaken at 200 rpm for 18 h. The optimal concentration of carbon source was determined with a series of carbon source concentrations (0.5%, 1%, 2%, 3%, 4%, 5%).

Nitrogen source: (NH_4_)_2_SO_4_, NH_4_Cl, urea, tryptone, peptone, yeast extract and beef extract (1%) were added to the fermentation medium as nitrogen sources, with the above fermentation conditions. The optimal concentration of nitrogen source was determined with a series of nitrogen source concentrations (0.5%, 1%, 2%, 3%, 4%, 5%).

Mineral salts NaH_2_PO_4_, K_2_HPO_4_, KCl, KH_2_PO_4_, MgSO_4_·7H_2_O, NaCl, MgCl_2_·6H_2_O and Na_2_HPO_4_ (100 mM) were added to the fermentation medium along with the optimized carbon and nitrogen sources under the same fermentation conditions as above. The optimal concentration of mineral salt was determined with a series of mineral salt concentrations (10 mM, 25 mM, 50 mM, 100 mM, 150 mM, 200 mM).

The amino acids Arg, Thr, Glu, Phe, Val, Gly, Ala and His (10 mM) were separately sterilized and added to the fermentation medium as amino acids, along with the corresponding optimal carbon and nitrogen sources and mineral salts, under the same fermentation conditions as above. The optimal concentration of amino acids in the medium was determined with a series of amino acid concentrations (2 mM, 5 mM, 10 mM, 20 mM).

The optical carbon source and nitrogen source were determined using the fermentation supernatant by absorbance at 297 nm. The optical mineral salts, amino acids and the concentration of carbon source, nitrogen source, mineral salts and amino acids were determined by HPLC.

**Screening of fermentation conditions to maximize andrimid production**

Inoculation amount and initial pH: BST187 was inoculated into 30 mL CB broth and supplemented with various seed cultures (0.1%, 0.5%, 1%, 2%, 3%) and varying pH values (6.0, 7.0, 7.8, 8.6) with the fermentation conditions described as above.

Fermentation times and temperatures: A 250 mL conical flask containing 100 mL of optimized CB media was inoculated with 1% seed culture and then incubated for different incubation periods (15, 18, 21 and 24 h at 28°C) and temperatures (18°C, 20°C, 24°C and 28°C for 18 h) on a rotary shaker (200 rpm).

Dissolved oxygen content: One hundred milliliters of optimized CB media was prepared in a modified conical flask (without baffle, triple baffle, quad baffle; Supplemental Fig. S3 a) and incubated with 1% seed culture at 28°C for 18 h at 200 rpm. The andrimid content was detected by HPLC.

**Supplemental Tables**

**Table S1.** The peak area of andrimid standard solution

| Numbers | Andrimid (mg/L) | Peak areas (mAU*S) | Purity (%) |
| --- | --- | --- | --- |
| 1 | 1000 | 57964.00 | 98.34% |
| 2 | 500 | 27544.60 | 99.48% |
| 3 | 100 | 5979.45 | 98.43% |
| 4 | 50 | 3280.88 | 100.00% |
| 5 | 10 | 653.20 | 100.00% |
| 6 | 5 | 352.92 | 100.00% |
| 7 | 1 | 197.01 | 100.00% |
| 8 | 0.5 | 91.83 | 100.00% |

**Table S2.** Composition of the candidate media evaluated within this study

| Cultivation Media | Constituent per L |
| --- | --- |
| Citrate-Beef (CB) | 20 g trisodium citrate dihydrate, 10 g beef extract, 10 g NaCl |
| King’s B Media (KB) | 10 g peptone, 15 g glycerol, 1.5 g K_2_HPO_4_, 6 g MgSO_4_ |
| Luria–Bertani (LB) | 10 g tryptone, 5 g yeast extract, 10 g NaCl |
| Terrific Broth (TB) | 12 g tryptone, 24 g yeast extract, 4.9 g glycerol, 2.31 g KH_2_PO_4_, 12.54 g K_2_HPO_4_ |
| Warkingsman (WA) | 5 g peptone, 3 g beef extract, 10 g glucose, 5 g NaCl |
| Nutrient Broth (NB) | 10 g peptone, 3 g beef extract, 5 g NaCl |

**Table S3.** Plackett-Burman experimental design and response value

| Std | Run | A**^*^** | B^*^ | C^*^ | D^*^ | E^*^ | F^*^ | G^*^ | Andrimid (mg/L) | Peak area  (mAU*S) |
| --- | --- | --- | --- | --- | --- | --- | --- | --- | --- | --- |
|  | 1 | -1 | +1 | +1 | +1 | -1 | +1 | +1 | 58.79 | 3434.19 |
| 9 | 2 | -1 | -1 | -1 | +1 | +1 | -1 | +1 | 70.94 | 4130.63 |
| 6 | 3 | +1 | +1 | +1 | -1 | +1 | -1 | +1 | 36.97 | 2183.04 |
| 10 | 4 | +1 | -1 | -1 | -1 | +1 | +1 | +1 | 83.27 | 4837.55 |
| 1 | 5 | +1 | -1 | +1 | -1 | -1 | +1 | -1 | 75.13 | 4371.02 |
| 12 | 6 | -1 | -1 | -1 | -1 | -1 | -1 | -1 | 22.90 | 1375.84 |
| 5 | 7 | +1 | +1 | -1 | +1 | +1 | +1 | -1 | 63.73 | 3717.29 |
| 4 | 8 | +1 | -1 | +1 | +1 | -1 | -1 | +1 | 64.62 | 3768.46 |
| 8 | 9 | -1 | -1 | +1 | +1 | +1 | +1 | -1 | 84.06 | 4882.83 |
| 3 | 10 | -1 | +1 | +1 | -1 | +1 | -1 | -1 | 11.89 | 744.71 |
| 2 | 11 | +1 | +1 | -1 | +1 | -1 | -1 | -1 | 30.99 | 1839.94 |
| 11 | 12 | -1 | +1 | -1 | -1 | -1 | +1 | +1 | 60.43 | 3528.02 |

**^*^:** A. Trisodium citrate dihydrate (g/L), B. Beef extract (g/L), C. MgCl_2_·6H_2_O (mM), D. Inoculation amount (%), E. Initial pH, F. Fermentation time (h), G. Fermentation temperature (℃)

**Table S4.** Results of Plackett-Burman experiment design

| NO. | Factors | Low level (-1) | High level (+1) | Stdized Effects | *P*-value |
| --- | --- | --- | --- | --- | --- |
| A | Trisodium citrate dihydrate (g/L) | 15 | 30 | 3.81 | 0.211 |
| B | Beef extract (g/L) | 15 | 30 | -11.51 | 0.011 |
| C | MgCl_2_·6H_2_O (mM) | 50 | 100 | -0.07 | 0.981 |
| D | Inoculation amount (%) | 0.5 | 1 | 6.88 | 0.055 |
| E | Initial pH | 6 | 7 | 3.17 | 0.284 |
| F | Fermentation time (h) | 21 | 27 | 7.19 | 0.048 |
| G | Fermentation temperature (°C) | 18 | 20 | 15.59 | 0.004 |

**Table S5.** Design and results of Box-Behnken experiment

| Std | Run | Beef extract (g/L) | Temperature (°C) | Fermentation time (h) | Andrimid (mg/L) | Peak area (mAU*S) |
| --- | --- | --- | --- | --- | --- | --- |
| 1 | 10 | 5 | 18 | 36 | 61.56 | 3592.62 |
| 2 | 13 | 25 | 18 | 36 | 85.83 | 4984.70 |
| 3 | 14 | 5 | 24 | 36 | 50.60 | 2964.23 |
| 4 | 9 | 25 | 24 | 36 | 42.08 | 2475.60 |
| 5 | 2 | 5 | 21 | 24 | 56.44 | 3299.49 |
| 6 | 3 | 25 | 21 | 24 | 49.90 | 2923.94 |
| 7 | 1 | 5 | 21 | 48 | 66.16 | 3856.35 |
| 8 | 12 | 25 | 21 | 48 | 78.29 | 4551.96 |
| 9 | 11 | 15 | 18 | 24 | 47.04 | 2760.28 |
| 10 | 15 | 15 | 24 | 24 | 44.40 | 2609.06 |
| 11 | 6 | 15 | 18 | 48 | 98.03 | 5683.78 |
| 12 | 5 | 15 | 24 | 48 | 47.09 | 2762.85 |
| 13 | 4 | 15 | 21 | 36 | 94.07 | 5456.99 |
| 14 | 8 | 15 | 21 | 36 | 93.69 | 5435.21 |
| 15 | 7 | 15 | 21 | 36 | 90.79 | 5268.72 |

**Supplemental Figures**


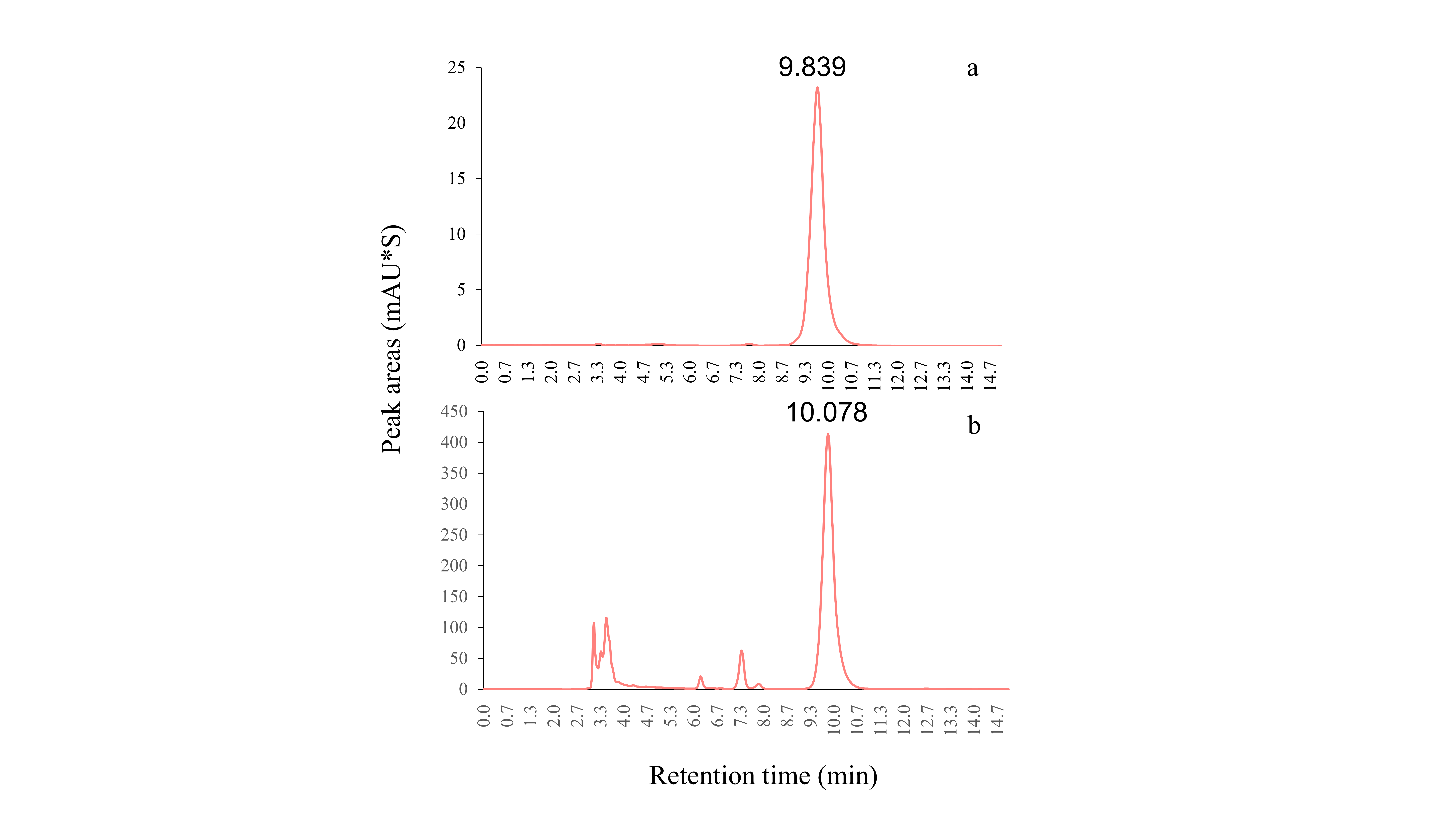


**Figure S1.** Analysis of HPLC chromatograms: standard of andrimid (**a.** RT=9.839; 0.01 mg/mL; 100%) and andrimid produced in BST187 (**b.** RT=10.078; 26.478%)


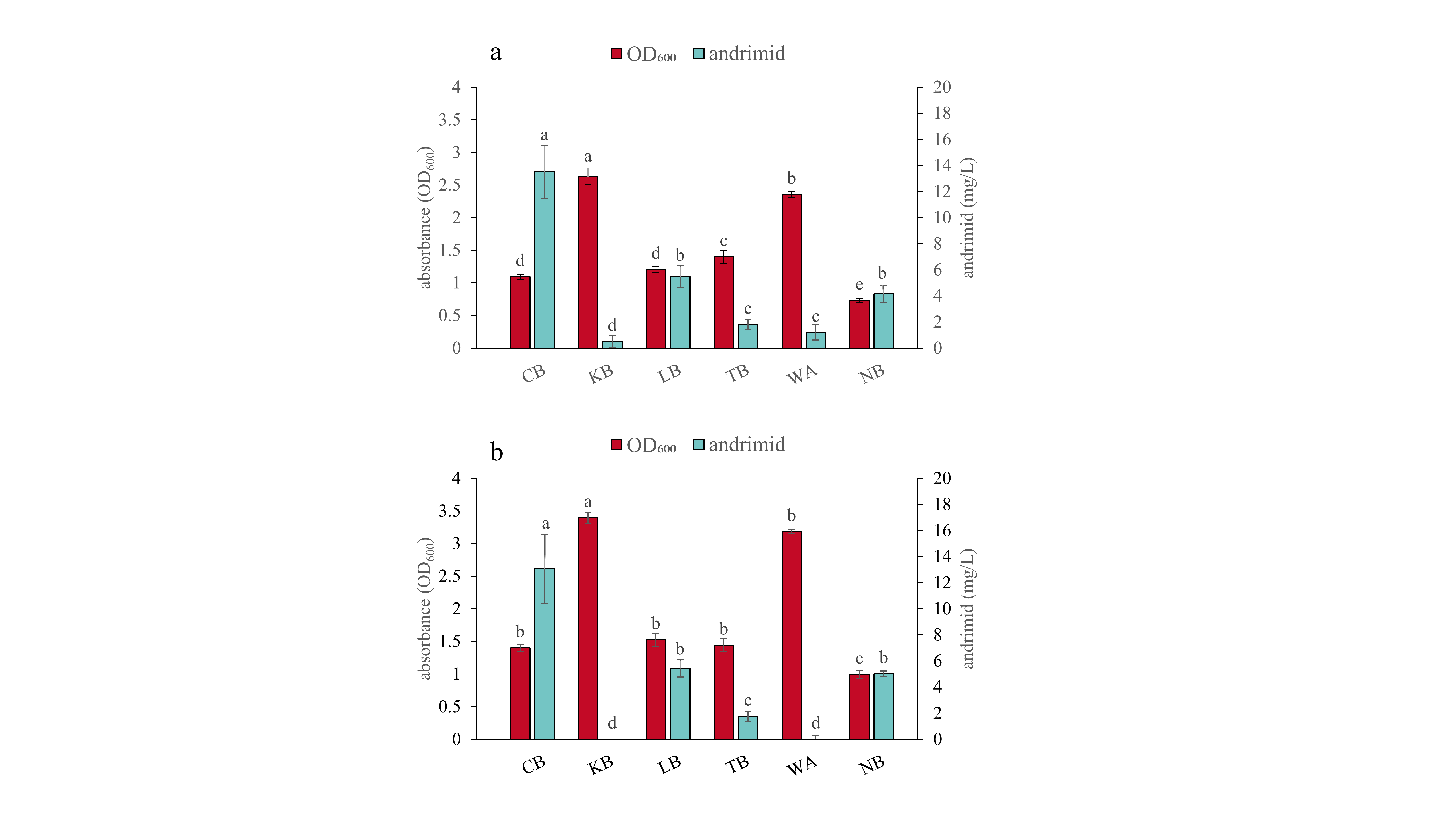


**Figure S2.** The biomass and andrimid production of BST187 in tested media; **a:** Cells were incubated in tested media for 18 h; **b:** Cells were incubated in tested media for 24 h; Citrate-Beef (CB), King’s B Medium (KB), Luria–Bertani (LB), Terrific Broth (TB), Warkingsman (WA), Nutrient Broth (NB); The different letters (a, b, c, d) represent the significant differences among the media (*p* < 0.05).


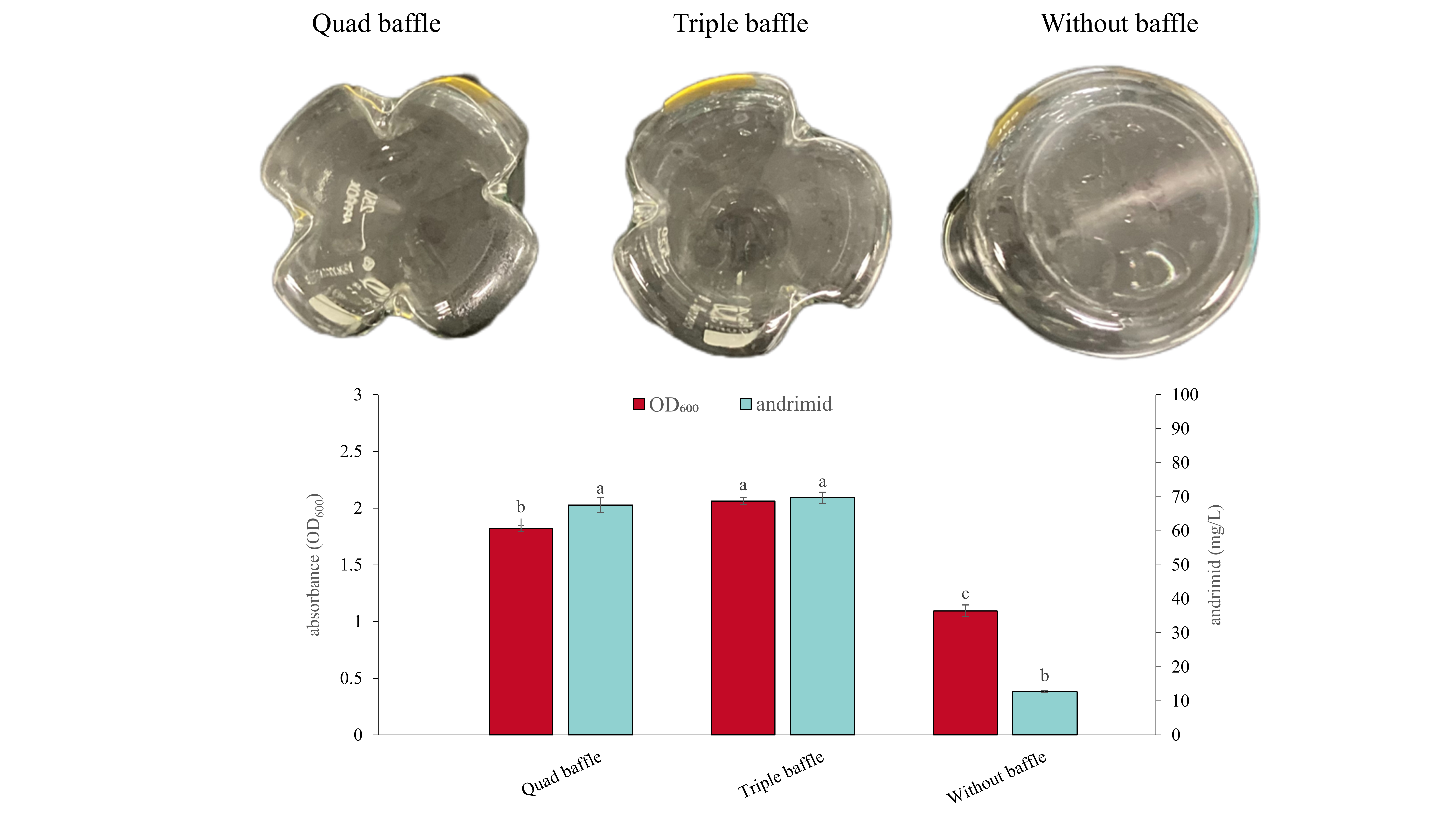


**Figure S3.** Effect of dissolved oxygen content on andrimid production; **a.** The type of conical flask; **b.** Effect of dissolved oxygen content on andrimid production by BST187; The different letters (a, b, c, d) represent the significant differences among the media (*p* < 0.05).


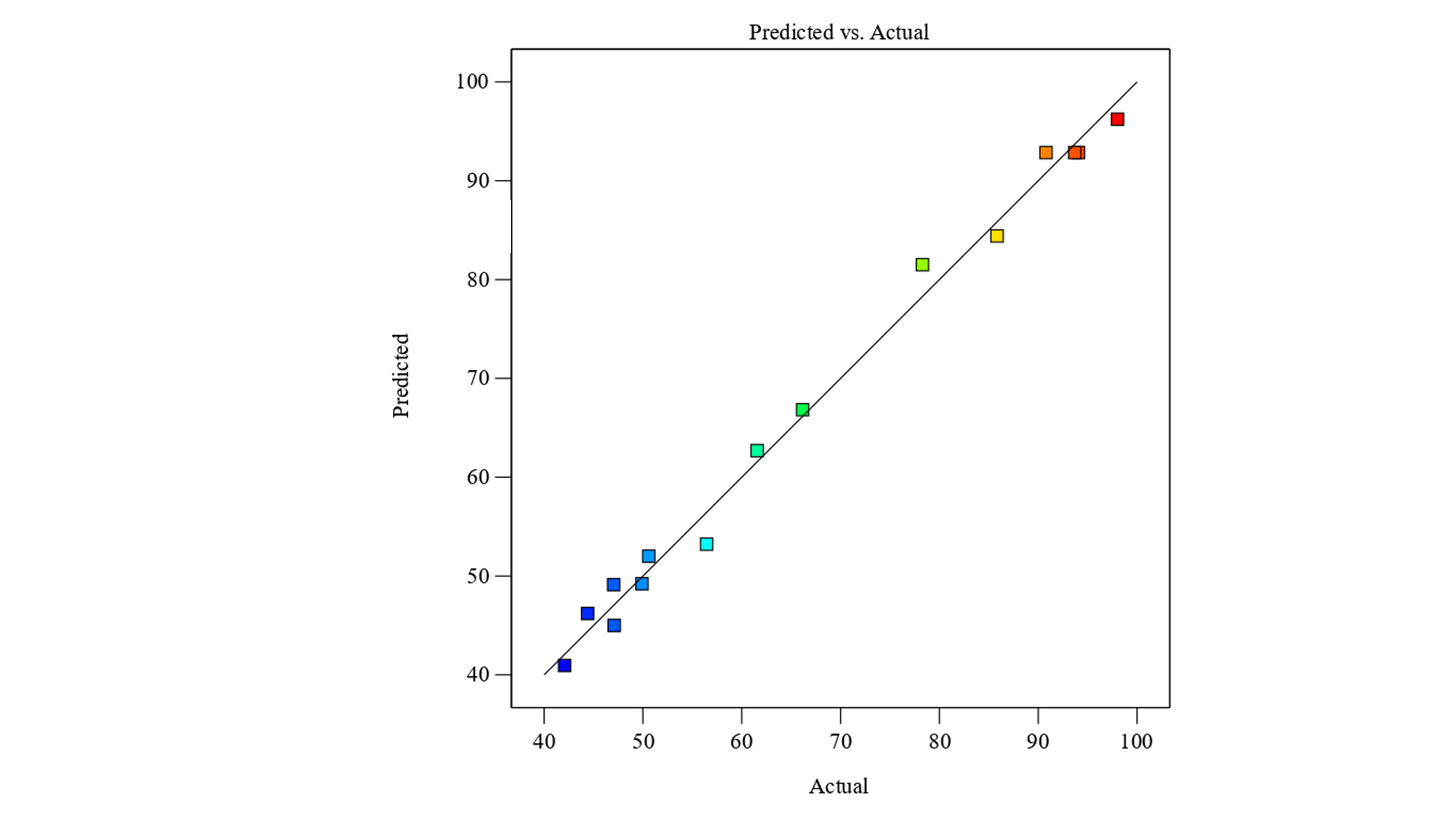


**Figure S4.** The correlation between the predicted and actual values for andrimid
